# Combining remote sensing and tracking data to quantify species' cumulative exposure to anthropogenic change

**DOI:** 10.1111/gcb.16974

**Published:** 2023-10-09

**Authors:** Claire Buchan, James J. Gilroy, Inês Catry, Chris M. Hewson, Philip W. Atkinson, Aldina M. A. Franco

**Affiliations:** ^1^ School of Environmental Sciences University of East Anglia Norwich UK; ^2^ CIBIO, Centro de Investigação em Biodiversidade e Recursos Genéticos, InBIO Laboratório Associado Universidade do Porto Vairao Portugal; ^3^ CIBIO, Centro de Investigação em Biodiversidade e Recursos Genéticos, InBIO Laboratório Associado, Instituto Superior de Agronomia Universidade de Lisboa Lisbon Portugal; ^4^ BIOPOLIS Program in Genomics Biodiversity and Land Planning, CIBIO Vairao Portugal; ^5^ British Trust for Ornithology, The Nunnery Thetford UK

**Keywords:** anthropogenic change, full season, migration, movement ecology, remote sensing

## Abstract

Identifying when and where organisms are exposed to anthropogenic change is crucial for diagnosing the drivers of biodiversity declines and implementing effective conservation measures. Accurately measuring individual‐scale exposure to anthropogenic impacts across the annual cycle as they move across continents requires an approach that is both spatially and temporally explicit—now achievable through recent parallel advances in remote‐sensing and individual tracking technologies. We combined 10 years of tracking data for a long‐distance migrant, (common cuckoo, *Cuculus canorus*), with multi‐dimensional remote‐sensed spatial datasets encompassing thirteen relevant anthropogenic impacts (including infrastructure, hunting, habitat change, and climate change), to quantify mean hourly and total accumulated exposure of tracked individuals to anthropogenic change across each stage of the annual cycle. Although mean hourly exposure to anthropogenic change was greatest in the breeding stage, accumulated exposure to changes associated with direct mortality risks (e.g., built infrastructure) and with climate were greatest during the wintering stage, which comprised 63% of the annual cycle on average for tracked individuals. Exposure to anthropogenic change varied considerably within and between migratory flyways, but there were no clear between‐flyway differences in overall exposure during migration stages. However, more easterly autumn migratory routes were significantly associated with lower subsequent exposure to anthropogenic impacts in the winter stage. Cumulative change exposure was not significantly associated with recent local‐scale population trends in the breeding range, possibly because cuckoos from shared breeding areas may follow divergent migration routes and therefore encounter very different risk landscapes. Our study highlights the potential for the integration of tracking data and high‐resolution remote sensing to generate valuable and detailed new insights into the impacts of environmental change on wild species.

## Graphical abstract

Pinpointing drivers of biodiversity decline is a major research priority; we develop a novel and widely applicable approach for quantifying risk exposure, combining high‐resolution risk mapping with tracking data. We demonstrate the conservation value of this method using the BTO's landmark decade‐long Common Cuckoo (*Cuculus canorus*) tracking project. Our approach reveals significant variation in risk exposure between stages of the annual cycle, and within and between migratory flyways. Cumulative risk exposure is also highly sensitive to phenology, with exposure in Cuckoos being greatest during winter, despite individuals typically wintering in lower‐risk areas.

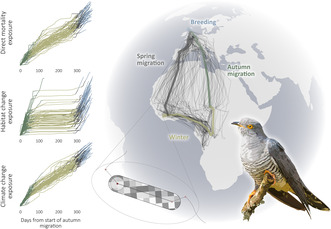

## 1 INTRODUCTION

Anthropogenic impacts associated with land‐use change and intensification, human settlements and infrastructure, and climate change are all linked to biodiversity declines (Bairlein, 2016; Kirby et al., 2008; Loss et al., 2015; Maxwell et al., 2016; Rigal et al., 2023), but their relative severity varies widely in space and time. Diagnosing the drivers of biodiversity declines requires understanding where organisms are most exposed to population‐limiting effects, and when in their life cycle these effects are greatest. Long‐distance migratory species are experiencing particularly severe population declines (Gregory et al., 2023; Laaksonen & Lehikoinen, 2013; Robbins et al., 1989; Rosenberg et al., 2019; Sanderson et al., 2006; Vickery et al., 2014; Yong et al., 2015), potentially because their reliance on spatially disparate seasonal resources increases their risk of encountering anthropogenic changes across the annual cycle (Newton, 2010; Robinson et al., 2009) and/or that migration may reduce the capacity for species to adapt to changing conditions on the breeding grounds (Flack et al., 2022; Møller et al., 2008). Recent research paints a complex picture regarding the importance of factors across seasonal stages, with different studies finding strong effects of wintering conditions (Adams et al., 2014; Davies et al., 2023; Kramer et al., 2018; Ockendon et al., 2012, 2014), breeding conditions (Morrison et al., 2013; Ockendon et al., 2013), and migratory conditions (Hewson et al., 2016; Lisovski et al., 2021; Studds et al., 2017), and we still lack sufficient understanding to confidently pinpoint and mitigate the key drivers of population declines among many migratory species.

Previous studies have typically sought to determine variation in exposure to anthropogenic change across the annual cycle from coarse knowledge of species' seasonal ranges (Buchan et al., 2022b; Kramer et al., 2018; Murray et al., 2018; Taylor & Stutchbury, 2016), or, more recently, geolocator data (Kramer et al., 2023). Though informative, many of these cannot account for within‐season and between‐individual spatiotemporal variability in site‐use, and they therefore lack sufficient resolution to quantify the relative contributions of different areas and seasons to change exposure. A spatiotemporally explicit approach can yield far greater insights, but this requires high resolution individual‐scale data on when and for how long individuals reside in particular locations, as well as their relative exposure to change at those locations. The last few decades have seen major advances in two parallel technological developments: bio‐logging/tracking and fine‐scale environmental remote sensing (Allan et al., 2018; Turner, 2014; Wassmer et al., 2020). Remote‐sensed data has revolutionised our capacity to undertake large‐scale mapping of anthropogenic change, in particular by facilitating the assessment and combination of multiple impacts (Buchan et al., 2022b; Halpern et al., 2008; Kennedy et al., 2019; Venter et al., 2016). At the same time, high‐resolution tracking data has transformed our understanding of individual‐level movements, yielding novel insights on migratory routes and timings (Davies et al., 2023; Hewson et al., 2016; van Bemmelen et al., 2019; Vansteelant et al., 2023), local movements (Shamoun‐Baranes et al., 2017), and survival (Buechley et al., 2021; Klaassen et al., 2014). To our knowledge, however, no studies have combined these two advances to measure year‐round individual exposure to remote‐sensed variables of anthropogenic change.

Here, we combine 10 years of tracking data for a declining long‐distance Afro‐Palaearctic migrant bird, the common cuckoo (*Cuculus canorus*, ‘cuckoo’ hereafter), with pan‐continental spatially explicit remote sensing data that allows us to measure individual‐level exposure to anthropogenic change across the annual cycle. We explore the relative contribution of metrics relating to direct mortality, habitat change and climate change to overall anthropogenic change exposure, in terms of both mean hourly change exposure and total accumulated change exposure accrued over the season. Variability in autumn migratory routes in the cuckoo has previously been linked to differences in survival on migration and population trends (Hewson et al., 2016), with birds following eastern flyways around the Mediterranean facing lower mortality rates during post‐breeding migration, and being more likely to breed at sites with positive population trends. We therefore also investigated how total accumulated change exposure scores varied between flyways during autumn migration and in the subsequent winter, and tested the relationship between non‐breeding season exposure and local‐scale population trends in breeding areas.

## 2 METHODS

### 2.1 Tracking data

We used data from 84 adult (second calendar year and older) male cuckoos tagged from 11 regions within the UK by the British Trust for Ornithology (May 2011 to February 2021), using Microwave Telemetry PTT‐100 tags (<5 g) following protocols outlined in Hewson et al. (2016). Tagging was carried out under approval from the Special Methods Technical Panel of the British Trust for Ornithology, in accordance with the ethical guidelines of the UK Government Home Office. Tags were programmed to a duty cycle of 10 h on, 48 h off (to allow for solar battery charging). Locations were obtained via the Argos satellite system and were then filtered following the methods detailed in Hewson et al. (2016) to the best‐quality location per duty cycle.

We separated individual cuckoo tracks into four stages of the annual cycle—autumn migration, Northern Hemisphere winter (‘winter’ hereafter), spring migration and breeding—using geographic and behavioral criteria (Soriano‐Redondo et al., 2020) following the methods of Hewson et al. (2016)—see Figure S1. As all birds were tagged during the breeding season, we started each bird‐year at the start of autumn migration, which we defined as the first movement of more than 50 km from the breeding location; these were individually checked and corrected to remove pre‐migratory movements. The end of autumn migration was defined as the first stopover south of 15° N, where a stopover is defined as three consecutive days during which the individual has a daily displacement of <50 km (Hewson et al., 2016). The wintering period ran from the end of the autumn migration to the start of the northward spring migration, in turn determined as the end of the last stopover south of 15° N. We defined the end of the northward migration to be the first fix within 50 km of the final breeding location; all fixes following this were assigned to the breeding season, which ended with the start of the following bird‐year autumn migration. Individuals that did not complete a season (e.g., due to death or tag failure) were excluded from that season but retained in those preceding. We characterized autumn migratory flyways for each bird‐year using the longitude at which the bird crossed latitude 35° N during the southward autumn migration, by which point the main migratory flyways across the Mediterranean have been determined.

### 2.2 Anthropogenic change surfaces

We used high‐resolution spatial layers adapted from Buchan et al. (2022b), in which metrics of anthropogenic change relevant to migratory birds are categorized as relating to direct mortality (roads (Meijer et al., 2018), nocturnal lights (NOAA, 2013), human population density (CIESIN, 2017), relative hunting threat (Buchan et al., 2022a), powerlines (Garrett, 2018; World Bank, 2017), windfarms (Dunnett et al., 2020), urbanization (Corbane et al., 2018)); habitat change (fertilizer use (FAO, 2019a; Klein Goldewijk et al., 2017), pesticide use (FAO, 2019b; Klein Goldewijk et al., 2017), conversion to urban land‐use); and climate change (temperature anomaly, temperature variability anomaly, precipitation anomaly, precipitation variability anomaly (Harris et al., 2020)). We did not include afforestation or conversion of land to cropland or pasture as potential risks, as cuckoos are considered able to exploit these habitats (Cramp et al., 1994). All layers represent relative change, with values between 0 (minimum measured change) and 1 (maximum measured change), and had a resolution of five arcminutes, with the exception of climate layers which were at 30 arcminutes, and thus did not capture spatial variability to the same extent as the other layers.

### 2.3 Estimating spatiotemporal change exposure

To determine each individual's temporal exposure to spatially varying anthropogenic change metrics, it was necessary to approximate the time spent within the areas through which it passed. We used the maximum range speed (*V*_mr_) of 10.4 m/s (39.96 km/h) calculated by Bruderer and Boldt (2001) using the formula developed by Pennycuick (1989) to estimate how long an individual cuckoo might reasonably take to cover the distance between fix locations. For each between‐fix track, we estimated the time spent flying (assuming a single hop) and assigned the remaining time as presence around the preceding fix location. Cuckoos are able to fly at ground speeds greater than the assumed *V*_mr_ of 39.96 km/h, particularly during migration (Bán et al., 2018); there were instances in our data where the time and distance between the fixes obtained indicated this must have occurred. In such cases, we assumed continuous flight between the fixes and calculated flight speed accordingly.

**FIGURE 1:**
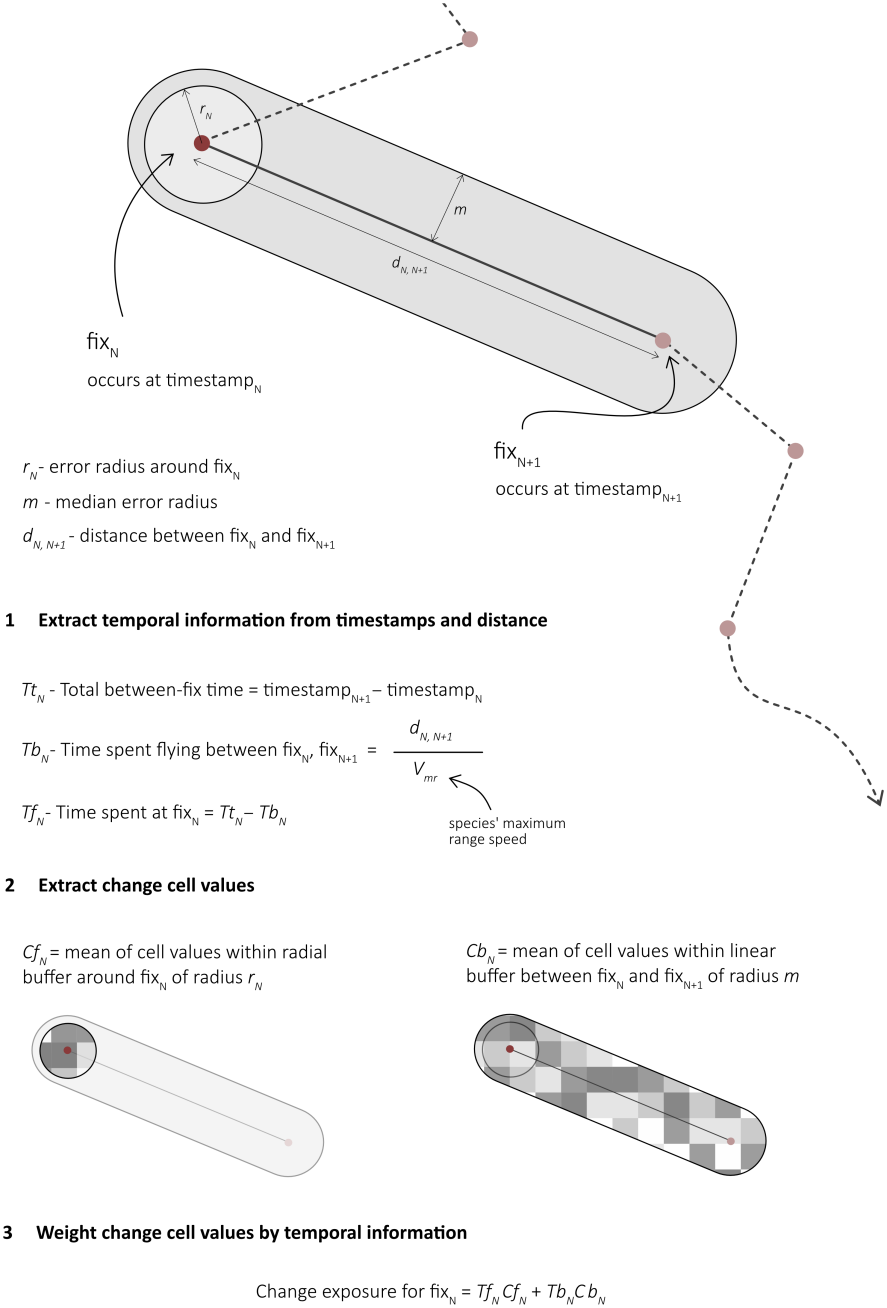
Schematic explaining the approach to combining tracking data and anthropogenic change surfaces to calculate per‐fix change exposure scores. These per‐fix scores subsequently feed into the calculation of mean change exposure and total accumulated change exposure. In the analysis presented here, *m* is equal to 258 m and *V*_mr_ is equal to 39.96 km/h (see main text).

To calculate change exposure at each fix, accounting for variable accuracy in fix locations given by the tags, we created a radial buffer around the location of a size determined by the per‐fix Argos error radius, and overlaid this onto each of the thirteen anthropogenic change surfaces—see Figure 1. We extracted the change surface values within this buffer and calculated the mean to yield the per‐fix radial buffer score (Cf_N_, Figure 1). To further account for uncertainty in the linearity of routes taken by birds between fixes, we used the median Argos error radius (258 m) to create a buffer around the straight‐line distance between each fix, and again extracted change layer values within this linear buffer and calculated their mean to yield the linear buffer change score (Cb_N_, Figure 1). We then multiplied the time in hours allocated to each fix (Tf_N_, Figure 1) by the extracted radial buffer change score, and the time allocated to the following between‐fix track (Tb_N_, Figure 1) by the extracted linear buffer change score, and summed these to calculate temporal exposure to each change surface for each ‘fix’. Finally, for each bird‐season (*n* = 239), we took the mean of these temporal exposure values to yield mean hourly change exposure, and the sum of the exposure values to yield total accumulated change exposure.

We used a combination of linear and fuzzy summation to account for likely correlated/non‐independent changes within each change type. This allowed us to combine the exposure values for each of the thirteen change surfaces into three composite change scores: change associated with direct mortality risks, habitat change, and climate change, following Buchan et al. (2022b)—see Supplementary Section S1. As the climate change anomaly layers are monthly, we extracted the climate change cell values for the layer corresponding to the month in which the bird passed through the cells.

### 2.4 Population change

To relate our exposure metrics to local‐scale rates of population change at breeding sites in the UK, we used data from the BTO Bird Atlas, which used volunteer surveyors across Britain and Ireland to create comprehensive distribution maps for two time periods: 1988–1991 and 2007–2011 (Balmer et al., 2014). Following Hewson et al. (2016), we calculated the standardized arithmetic difference between cuckoo abundance in 1988–1991 and in 2007–2011, where abundance is the proportion of 2‐km Atlas‐surveyed squares within each 10‐km National Grid square that contained cuckoos. Sampled cuckoos were tagged at eleven different breeding sites, and we created a 25‐km radius buffer around each capture location and merged these to create polygons representing the spatial footprint of each sampled site (Figure S2). We then extracted the abundance change for each 10 × 10 km grid square within each footprint, and calculated a mean abundance change for each site, with values for each grid square weighted by the min‐max scaled area of overlap with the footprint polygon (such that grid squares partially outwith the polygon were proportionally downweighted).

### 2.5 Analysis

We used linear mixed‐effects models to quantify differences in log mean hourly change exposure and log accumulated change exposure per bird‐season (*n* = 239) between stages of the annual cycle, analyzing each change type separately (direct mortality, habitat change and climate change) and including individual bird ID (*n* = 53) as a random effect. We used likelihood ratio tests to assess the significance of season as a categorical predictor, and post‐hoc multiple comparison tests to assess significance of between‐season differences.

To assess the influence of autumn flyway on change exposure during autumn and the subsequent winter, we modelled log accumulated change exposure for each of the three change types at each stage as a function of track longitude at 35° N. We compared the fit of linear models and generalized additive models (thin plate regression splines) with default selection of smoothing parameters to explore whether there was statistical support for non‐linear relationships; we used *r* ^2^ values and AIC values to identify best fitting models. We also modelled how winter accumulated change exposure varied with the longitude of the first winter fix (rather than at 35° N) to capture variability with respect to flyway destination.

To test whether seasonal change exposure scores were related to breeding site population trends, we calculated the mean change exposure experienced by individuals from each breeding site (*n* = 11) for each season (*n* = 10 for the breeding season) and used univariate linear models to assess the influence of mean per‐season exposure on mean site abundance change.

We repeated all analyses on a subset of the dataset with any bird‐season with a between‐fix gap of more than 10 days and greater than 2000 km (e.g., due to low device battery) removed (*n* = 17) to ensure results were not sensitive to the inclusion of these relatively data‐poor tracks. We conducted all analyses in R version 4.0.3 (R Core Team, 2020), using packages {raster} (Hijmans et al., 2022) and {sf} (Pebesma, 2018) for manipulation of spatial data, {mgcv} (Wood, 2011) and {lme4} (Bates et al., 2015) for creation of generalized additive models and linear mixed effects models respectively, and {multcomp} for conducting post‐hoc comparisons (Hothorn et al., 2008). In all cases, we assessed significance of univariate predictors using likelihood ratio tests using the maximum likelihood estimator for standard deviation of errors, using {lmtest} (Zeileis & Hothorn, 2002) at *α* = .05, using Bonferroni adjusted *P*‐values to control for family‐wise error rate in analyses with multiple tests. All continuous variables were z‐score scaled, and, where necessary, log‐ or cube‐transformed to ensure normality of model residuals; see Supplementary Section S2 for details of full model structures.

## 3 RESULTS

**FIGURE 2:**
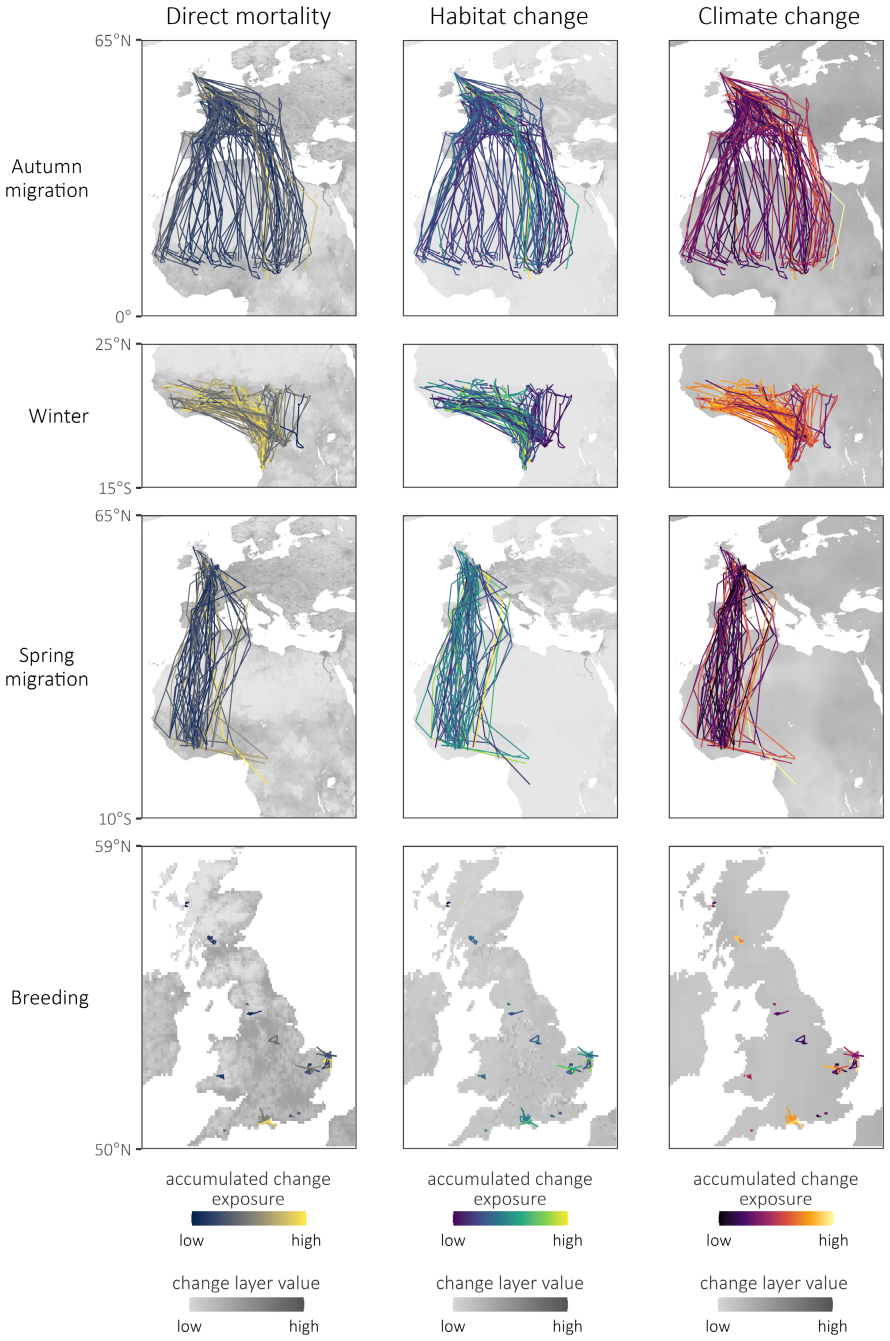
Tracks of common cuckoos throughout the annual cycle; each line represents a bird‐year—seasonal sample sizes are given in Table S1. Lines are colored according to total accumulated within‐season exposure to change (colours standardized within seasons). Lines are overlaid onto the change layers from which exposure scores are calculated, except in the case of climate, for which the basemaps show the mean of the relevant months.

Filtering the initial tracking dataset of 84 tagged cuckoos left a sample of 53 individuals that met our criteria, yielding 239 bird‐seasons from 86 bird‐years (Figure 2; Table S1). Spring migration was the shortest of the four annual stages, accounting for approximately 7% of the year on average, while winter was the longest stage, accounting for c. 63% of the year.

### 3.1 Between‐season exposure

**FIGURE 3:**
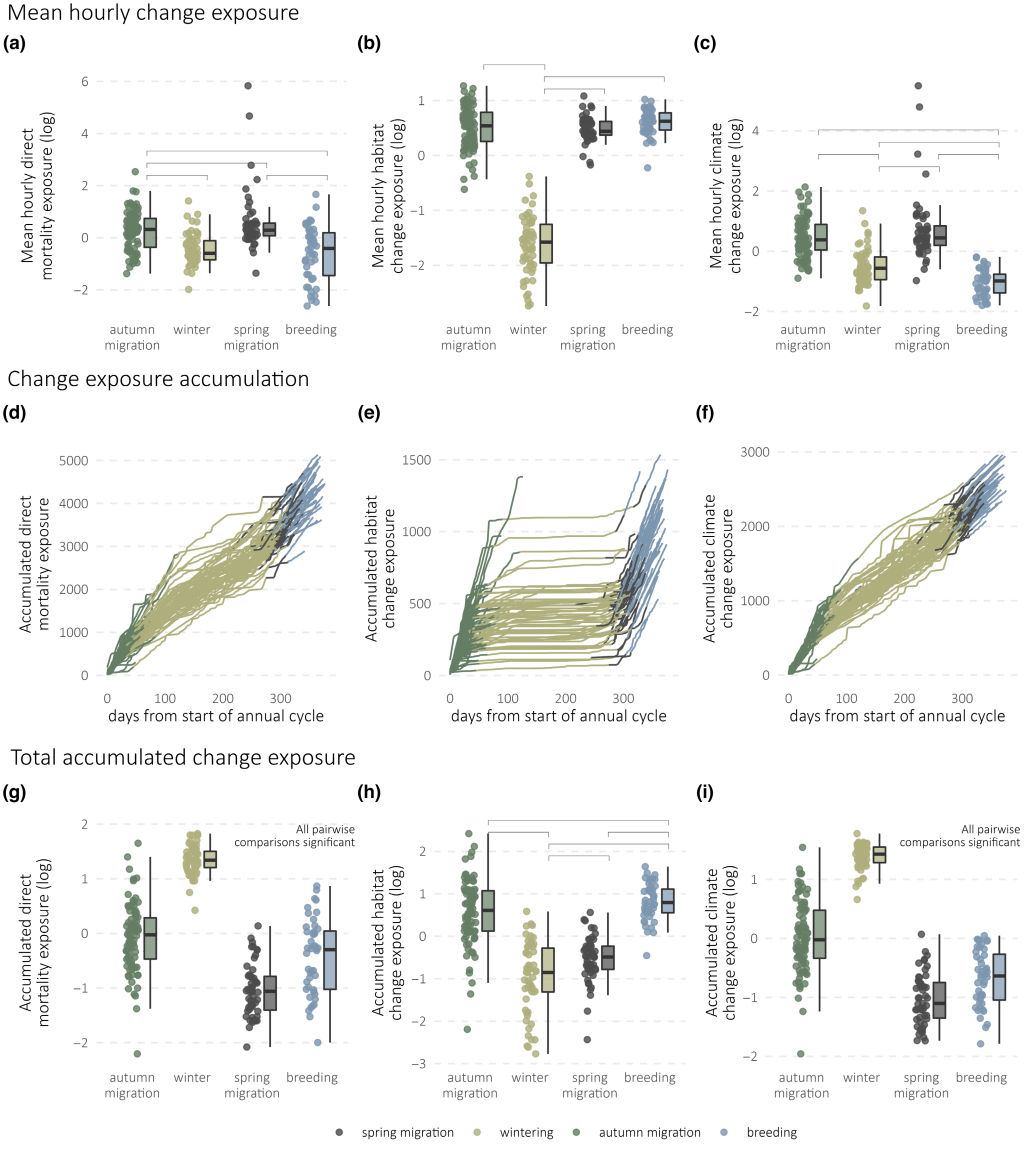
Patterns of mean change exposure (a–c), change exposure accumulation (d–f) and total accumulated exposure (g–i) across seasonal stages. Boxplots in (a–c) and (g–i) show the median value, interquartile range and Tukey‐style whiskers, while points show raw mean change exposure data for each bird‐season. Lines in (d–f) show bird‐years as they progress throughout the annual cycle, where the *x*‐axis represents time elapsed since the start of autumn migration, while the *y*‐axis values represent cumulative time‐weighted exposure for each change type. Results of pairwise comparisons are given in Table S2; square brackets indicate significant between‐season pairwise comparisons.

Seasonal stage explained between 21% and 85% of variance in mean hourly exposure, and between 53% and 78% of variance in accumulated exposure, across the three change types (Table 1). Mean hourly direct mortality and climate change exposure scores were significantly greater during the spring and autumn migrations than during the breeding and wintering seasons (Figure 3a; Table S2), but the accumulated change exposure for both was significantly higher in the winter stage than any other (Figure 3g; Table S2). In the case of habitat change, mean hourly exposure was lowest in the winter compared to all other seasons (Figure 3b; Table S2), and accumulated exposure was highest in autumn, followed by spring migration, and lowest in winter (Figure 3h; Table S2). The breeding season saw the lowest mean hourly climate change exposure compared to all other seasons, followed by the winter (Figure 3c; Table S2). However, accumulated exposure to climate change was highest during winter (Figure 3i)—significantly higher than any other season (Table S2). For both mean hourly and accumulated exposure scores, sensitivity analyses run on a subset of the dataset with 17 data‐deficient bird‐seasons removed yielded similar results in all cases (Appendix: Tables A1 and A2).

**TABLE 1:** Summaries of six univariate linear mixed‐effects models and associated likelihood ratio tests assessing the effect of season on mean hourly change exposure and accumulated change exposure for the three change types, with bird identity as a random effect.

Response variable		Marginal *r* ^2^	Likelihood ratio test statistics		
Metric	Change type		*χ* ^2^	*χ* ^2^ df	*p*‐value
Mean hourly exposure	Direct mortality	.21	57.81	3	<.001
	Habitat change	.85	461.72	3	<.001
	Climate change	.44	157.66	3	<.001
Accumulated exposure	Direct mortality	.70	284.32	3	<.001
	Habitat change	.53	176.97	3	<.001
	Climate change	.78	350.33	3	<.001

*Note*: Post‐hoc tests of pairwise comparisons are given in Table S2. Marginal *r* ^2^ for linear mixed‐effects models calculated following Nakagawa and Schielzeth (2013).

### 3.2 Migratory route

**FIGURE 4:**
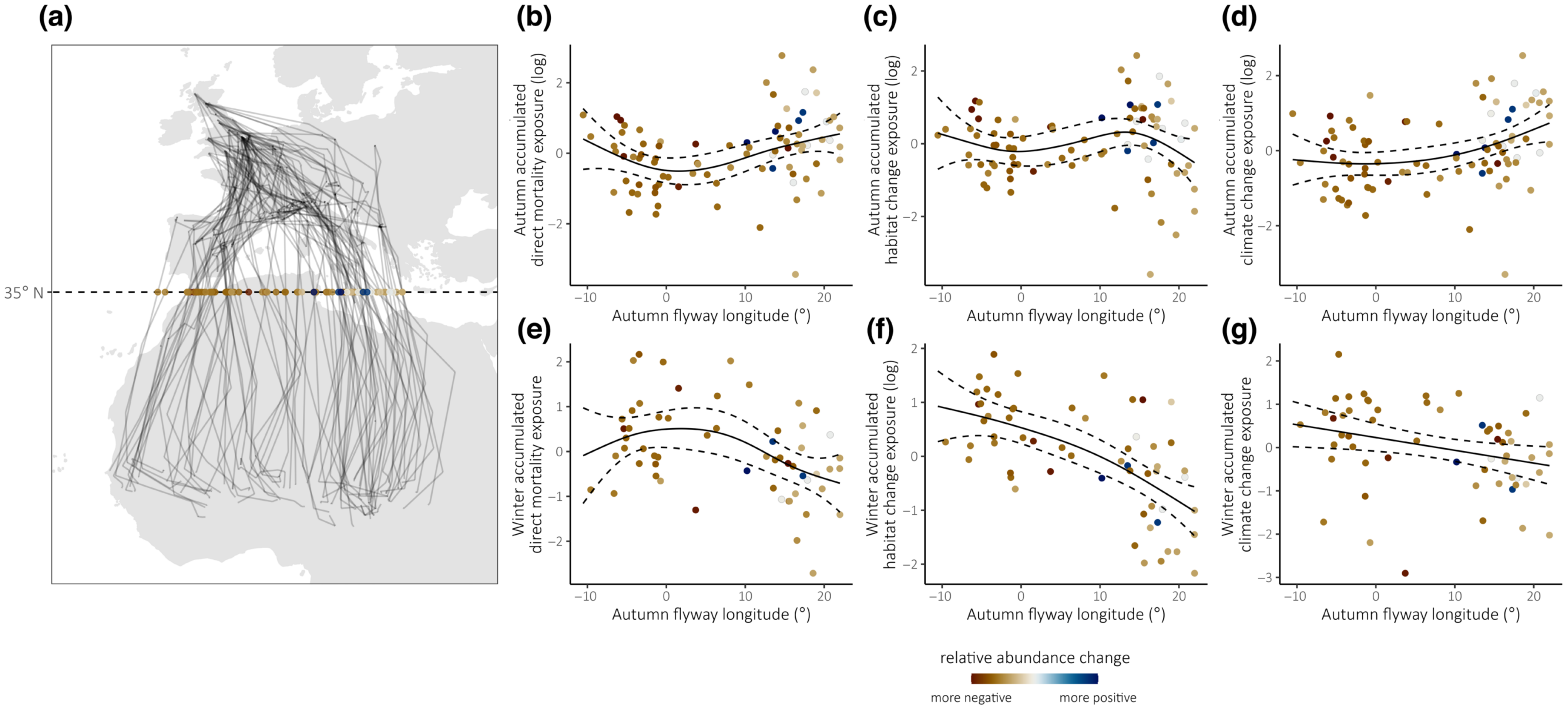
Patterns of total stage‐specific change exposure scores with respect to autumn flyways. Autumn migratory routes of common cuckoos (a) are characterized by the longitude at which they cross 35° latitude (points). Scatterplots show the relationship between autumn flyway longitude and metrics of autumn (b–d) and winter (e–g) accumulated exposure to the three change types, where points show raw bird‐season data and solid and dashed lines indicate model‐predicted means and 95% confidence intervals respectively from the most parsimonious models (see Table S3). Points are coloured according to the relative population abundance change at the breeding site of each individual (see Section 2).

We found significant variation in accumulated change exposure across the east–west axis of migratory flyways for direct mortality and climate change in the autumn, and for all change types in the winter (Table S3), but this variation was generally non‐linear (Table S3; Figure 4b–g). Accumulated exposure to changes associated with direct mortality risks during autumn migration tended to be higher in routes towards either longitudinal extreme (Figure 4b), whereas both climate change and habitat change exposure scores were higher during autumn along more easterly routes (Figure 4c,d). Accumulated exposure to all three change types in the subsequent wintering period were lower among individuals that took more easterly routes during the autumn (Figure 4e–g). Although performance was in some instances similar, generalized additive models fit the data better than linear models in all cases except for winter climate change exposure, for which the linear model fit better (Table S3). Sensitivity analyses excluding data‐deficient bird‐seasons yielded similar results (Appendix: Table A3), as did analyses using initial winter longitude as a measure of flyway route (Appendix: Table A5; Figure A1).

### 3.3 Breeding site abundance change

We found no significant relationships between local population abundance change around our sampled breeding sites and mean seasonal accumulated change exposure scores, with the exception of breeding season direct mortality (Figure S3), where greater exposure was associated with lower population trends, explaining 72% of the variation in breeding site population abundance change (Table 2). These results persisted in the sensitivity analyses (Appendix: Table A4). Examination of the migratory tracks in this dataset indicates relatively weak migratory connectivity in UK‐breeding common cuckoos (Figure S4), such that our sample for each breeding site contains individuals with spatially divergent migrations and very different change exposure levels. It is therefore unsurprising to find only a correlation between mean site‐level breeding season change exposure and breeding site population trends, as site‐level means mask considerable within‐site variation in non‐breeding season exposure to change (see Section 4).

**TABLE 2:** Model summaries of nine univariate models and associated likelihood ratio tests assessing the effect of mean seasonal accumulated change exposure for birds from each of the 11 sites on the mean abundance change per site. Bold font indicates statisitcal significance after Bonferroni correction.

Predictor variable		Intercept	*β*	*r* ^2^	Sample size	Likelihood ratio test statistics			
Season	Change type					*χ* ^2^	*χ* ^2^ df	*p‐*value	Bonferroni‐corrected *p*‐value
Autumn migration	Direct mortality	−0.19	.10	.17	11	2.11	1	.147	1.000
	Habitat change	−0.19	.09	.13	11	1.50	1	.221	1.000
	Climate change	−0.19	.09	.14	11	1.69	1	.194	1.000
Winter	Direct mortality	−0.19	−.6	.05	11	0.61	1	.434	1.000
	Habitat change	−0.19	−.12	.22	11	2.79	1	.095	1.000
	Climate change	−0.19	−.02	.01	11	0.07	1	.793	1.000
Spring migration	Direct mortality	−0.19	−.02	.01	11	0.08	1	.776	1.000
	Habitat change	−0.19	.08	.10	11	1.14	1	.286	1.000
	Climate change	−0.19	−.06	.06	11	0.68	1	.410	1.000
**Breeding**	**Direct mortality**	**−0.18**	**−.22**	**.72**	**10**	**12.75**	**1**	**.0004**	**.024**
	Habitat change	−0.18	−.20	.59	10	8.99	1	.003	.108
	Climate change	−0.18	−.02	.01	10	0.07	1	.789	1.000

## 4 DISCUSSION

Our study showcases how the integration of remote‐sensed data products with satellite tracking can generate detailed new insights into the ways organisms are influenced by anthropogenic change. Among long‐distance migrants, where exposure to anthropogenic change may vary hugely between individuals and populations across the annual cycle, this approach has the potential to deliver significant advances in our understanding of spatiotemporal threat exposure, and in turn to inform the spatiotemporal targeting of conservation efforts. By linking individual movement patterns to spatial change variation at high temporal resolution, our approach revealed that accumulated change exposure in our study species was generally highest during their winter life stage, despite the areas occupied during this season having undergone relatively lower levels of anthropogenic change than other parts of the species range (Figure 3a–c; Buchan et al., 2022a). Our results highlight the complexity of spatial variation in change exposure within and between migratory flyways, demonstrating the critical role migratory route choice plays in shaping the potential risks faced by individuals across the annual cycle (Hewson et al., 2016).

### 4.1 Between‐season differences in change exposure

We found that proximate levels of hourly exposure to impacts relating to direct mortality and climate change were highest during the two migration phases for cuckoos. This likely reflects the relatively high levels and intensity of anthropogenic transformation seen in the areas cuckoos traverse on migration (IPCC, 2013; Venter et al., 2016), and supports the notion of ‘multiple jeopardy’ incurred by migrants during the mobile stages of their life cycle (Gilroy et al., 2016). However, accounting for the longer duration of the wintering phase in our study species (Table S1; Figure 3d–f) reveals that the winter stage contributes the most to accumulated exposure to direct mortality changes and climate change. As brood parasites, cuckoos have particularly short breeding seasons (Table S1), exacerbating the discrepancy between mean hourly and accumulated breeding season change exposure we report here (Figure 3). Species‐specific ecological drivers of phenology (e.g., reproductive and moult strategies) (Thorup et al., 2007), in addition to responses to changes in climatic cues (Lehikoinen et al., 2004), may therefore also be important in determining how species vary in their relative accumulated change exposure between seasons.

Across all three types, accumulated exposure to anthropogenic change was generally higher on autumn migration than spring migration (Figure 3g–i). The more easterly migratory routes used in the autumn tend to have greater hourly exposure levels than the westerly routes (Figure S5), possibly because a greater proportion of these routes lie within Europe, where the intensity and extent of anthropogenic change is highest (Figure 2). All sampled birds returned during spring via the more westerly flyway (Figure 2), which may in turn explain the lower exposure levels on spring migration. The discrepancy in accumulated change exposure seen between spring and autumn is also in part driven by the longer duration of autumn migration (Table S1). Generally, the pace of spring migration is thought to be driven by time constraints, while that of autumn migration is driven by resource availability, with longer stopover durations and slower flight speeds in the latter (Nilsson et al., 2013)—yielding greater exposure to anthropogenic change.

Levels of exposure to habitat change were highest in the autumn migration and breeding seasons (Figure 3h; Table S2). This largely reflects macro‐scale spatial patterns of urbanization and agrochemical use, which are in general markedly lower in the west and central African wintering grounds of our focal species than elsewhere in their annual range (Figure 3b). In other species, patterns of exposure to changes associated with habitat are likely to vary significantly in relation to habitat specialism—for instance, species unable to utilise agricultural land‐use types (unlike, to some extent, cuckoos) may be particularly exposed to habitat changes in sub‐Saharan areas, where agricultural expansion has been rapid. Indeed, cuckoos have complex habitat requirements driven by host availability in the breeding season, as well as microhabitats for foraging and roosting (Denerley et al., 2019; Stokke et al., 2007), with evidence that cuckoos prefer mosaic semi‐open habitats in the non‐breeding season (Williams et al., 2016), complicating the relationship between land cover changes and realized risks to fitness. Species‐specific refinement of spatial change maps to capture key stressors likely to be most relevant for particular species may further enhance the potential for this approach to identify important spatiotemporal patterns.

Our finding of higher accumulated change exposure in the winter than during the two migratory phases is perhaps surprising, given evidence from other species that mortality rates tend to peak during migration (Klaassen et al., 2014; Oppel et al., 2015; Sergio et al., 2019; Sillett & Holmes, 2002). It is important to note that our approach focusses on exposure to anthropogenic change, but does not capture underlying survival risks that are largely independent of change, such as ocean and desert barriers, exposure to unfamiliar habitat and increased predation susceptibility. The migratory phases for trans‐Saharan migrants are likely to be inherently risky due to the added presence of these threats and may therefore be expected to have higher baseline levels of mortality risk compared to other seasonal stages regardless of human impacts. Extending our approach to account for these ‘baseline’ risks could yield further insights, and allow for the consideration of seasonal carry‐over effects from previous exposure and their potential to influence survivorship during more inherently risky migratory stages.

Survivorship bias may also play a role in influencing our findings, as we excluded incomplete bird‐seasons that may have arisen due to mortality. Bias could occur if, in the cases where tag death represents true bird death, the censored tracks moved through particularly high‐change exposure areas. That we have only data for male cuckoos may also introduce bias if there are between‐sex differences in survival or migratory strategy (Briedis et al., 2019), which have not been studied in cuckoos. Additionally, although the change layers included here are tailored to the cuckoo as a long‐distance, habitat generalist migrant, measurements of accumulated change exposure represent only one element of vulnerability to change, which can be defined as a combination of exposure, sensitivity, and capacity to respond (Foden et al., 2013). Detailed assessment of the sensitivity—which can variously be influenced by, for instance, degree of specialism, fragile interspecific reliance, morphological traits (Buchan et al., 2022b; Foden et al., 2013; Mason et al., 2019)—of the target species to each anthropogenic change layer will complement exposure metrics. Similarly, our approach does not capture the capacity for fine‐scale behavioral adaptation to risks, such as micro‐avoidance (Everaert, 2014; Plonczkier & Simms, 2012), nor the extent to which individuals are risk‐naïve or ‐omniscient (Klaassen et al., 2006). Indeed, risk perception and avoidance may be context‐dependent (Sol et al., 2018), and may itself have negative fitness consequences (Doherty et al., 2021). Incorporating risk avoidance into exposure quantification, particularly by modelling individual movements at even higher spatiotemporal resolutions, may be especially valuable for understanding exposure during active movement phases. Avoidance behaviors themselves could potentially be detected through pattern analysis of high‐resolution tracking data (including accelerometer data) to detect movement responses in relation to proximate threats.

### 4.2 Between‐flyway differences in change exposure

Across the two broad flyways used by cuckoos, we found that patterns of anthropogenic change exposure during autumn were spatially complex, with no clear flyway‐scale differences in overall exposure during the migration phase itself (Figure 4b–d). However, across all three change types, accumulated exposure in the subsequent winter was significantly lower for individuals whose autumn migratory flyway was further east (Figure 4d–f). This agrees with Hewson et al. (2016), who previously found that site‐level breeding population trends were positively correlated with the proportion of tagged cuckoos that take more easterly autumn migration routes. This connection implies that conditions experienced during the winter phase—in addition to or in synthesis with those experienced during autumn migration—may be of particular demographic importance, although it is not possible here to disentangle the extent to which local population trends may also be driven by breeding site effects (Buchan et al., 2021; Morrison et al., 2013). Our findings do not align with the results of Hewson et al. (2016) regarding flyway survival, who reported greater mortality during migration for birds taking western routes, while we find no consistent between‐flyway differences in exposure to anthropogenic changes during autumn. This may again reflect the inherent riskiness of some routes independent of anthropogenic change (see above), or that the human impacts captured by our change surfaces are not demographically relevant for this species. This discrepancy may also reflect the possibility that survival during migration is also influenced by carryover effects of breeding conditions (Harrison et al., 2011; Hewson et al., 2016), or a role of survivorship bias in our sample.

### 4.3 Breeding site abundance change

We found no relationship between average change exposure scores across non‐breeding stages and recent abundance trends at the breeding site level. However, direct effects of migratory change exposure on site‐level breeding population change would only be expected in populations that exhibit strong migratory connectivity, such that the individuals inhabiting a breeding site follow similar migrations and therefore have similar non‐breeding experiences. Migratory connectivity in our study population is comparatively weak (Figure S4), reducing the likelihood of finding a relationship between mean site‐level accumulated change exposure and population trends, as site‐level means mask considerable variation in non‐breeding experiences among cuckoos that breed at the same site. Similarly, although the population change data we use is best available for the scale of our study, it represents a period leading up to—but not including—the years of tracking data. Links between change exposure and population change would rely on temporally consistent migratory connectivity, such that the cuckoos contributing to the population abundance data had similar exposure levels to the tagged individuals included in our analysis. Extending our approach to assess how individual change exposure patterns relate to levels of risks, threats or even opportunities experienced, and therefore how they influence individual‐level demographic parameters—breeding success, survival and body condition—would likely yield important new insights into the effects of anthropogenic change exposure on this species and other migratory birds.

## 5 CONCLUSION

Combining remote‐sensed environmental information with tracking data represents a powerful means of assessing how organisms accumulate exposure to environmental change, bringing an important step towards better quantification of exactly where and when organisms are most exposed to potential anthropogenic threats. Our results reinforce the importance of full annual cycle approaches (Marra et al., 2015), and of exploring between‐season temporal variability in anthropogenic impacts. The increasing availability of high spatial and temporal resolution tracking data and remote‐sensed environmental data will enable more accurate estimation of change exposure in future; combining this with individual‐level data on condition, survival and breeding success will shed new light on the demographic impacts of anthropogenic change for migratory birds, and help pinpoint key areas and issues for targeted conservation action. By better elucidating where and when individuals are exposed to threats, future studies may also gain more power in understanding species' sensitivity to those threats—which can further enhance strategic conservation planning.

## AUTHOR CONTRIBUTIONS

**Claire Buchan:** Formal analysis; methodology; visualization; writing – original draft. **James J. Gilroy:** Conceptualization; funding acquisition; supervision; writing – review and editing. **Inês Catry:** Supervision; writing – review and editing. **Chris M. Hewson:** Data curation; funding acquisition; resources; writing – review and editing. **Philip W. Atkinson:** Data curation; supervision; writing – review and editing. **Aldina M. A. Franco:** Conceptualization; funding acquisition; supervision; writing – review and editing.

## CONFLICT OF INTEREST STATEMENT

The authors declare no conflicts of interest.

## Supporting information


Appendix S1

Data S1

## Data Availability

The data underpinning the analyses presented here are openly available from Figshare at https://doi.org/10.6084/m9.figshare.23576031 (Buchan et al., 2023), and which were calculated using publicly available risk surfaces listed in Buchan et al. (2022b).
