# Supplementary material for: Combining remote sensing and tracking data to quantify species' cumulative exposure to anthropogenic change
Source: Glob Chang Biol. 2023 Oct 9;29(23):6679–92. doi: 10.1111/gcb.16974 (PMC10946810; doi:10.1111/gcb.16974)
Supplement: Supplementary file 1 — Appendix S1 [file GCB-29-6679-s001.pdf]

Combining remote sensing and tracking data to quantify species' cumulative exposure to anthropogenic change

Claire Buchan*, James J. Gilroy, Inês Catry, Chris M. Hewson, Philip W. Atkinson, Aldina M. A. Franco

Appendix 1

This document describes analyses additional to those reported in the above manuscript, and consists of the following materials:

Sensitivity analyses

Table A1 Summaries of linear models assessing the effect of season on mean hourly and accumulated change exposure, conducted on a reduced dataset with data-poor tracks removed

Table A2 Post-hoc tests of multiple comparisons carried out on models assessing effect of season on mean hourly and accumulated change exposure, conducted on a reduced dataset with data-poor tracks removed

Table A3 Summaries of linear and generalized additive models assessing the influence of autumn flyway longitude on autumn and winter accumulated change exposure, conducted on a reduced dataset with data-poor tracks removed

Table A4 Summaries of univariate models assessing the effect of mean accumulated change exposure on mean population abundance change per site, conducted on a reduced dataset with data-poor tracks removed

Winter destination

Table A5 Summaries of linear and generalized additive models assessing the influence of initial winter longitude on metrics of accumulated winter change exposure

Figure A1 Model-predicted relationships between initial winter longitude on metrics of accumulated winter change exposure

References cited

Sensitivity analyses

When processing cuckoo tracking data, we flagged any bird-seasons featuring a between-fix gap longer than 10 days and greater than 2000 km, as the inferred path between these fixes is unlikely to be accurate. Seventeen of the 239 fixes (7%) fit these criteria. We re-ran all analyses reported in the main text on a subset of the full dataset, with the seventeen data-poor bird-seasons excluded.

In all cases, inference remained similar to that of the results run on the full dataset. Mean hourly change exposure and accumulated change exposure both varied with stage of the annual cycle (Table A1), with post-hoc tests revealing the same seasonal patterns of exposure (Table A2). The effect of migratory longitude on autumn and winter accumulated change exposure scores remained similar, although when conducted on the dataset without the data-poor birds there was no statistical support for an effect of migratory longitude on winter climate change exposure (Table A3). There was again no statistical support for any effect of accumulated change exposure in the non-breeding seasons on population abundance change, and support for a negative effect of breeding season direct mortality exposure on population change (Table A4).

Table A1 – Summaries of six univariate linear mixed-effects models and associated likelihood ratio tests assessing the effect of season on mean hourly exposure and accumulated exposure for the three change types, with bird identity as a random effect, run on a subset of data excluding data-poor bird-seasons. Marginal r^2 for linear mixed-effects models calculated following Nakagawa & Schielzeth (2013).

Response variable			Likelihood ratio test statistics		
Metric	Change type	Marginal r^2	χ^2	χ^2 df	*P*-value
Mean hourly exposure	direct mortality	0.21	54.12	3	< 0.001
	habitat change	0.87	445.85	3	< 0.001
	climate change	0.53	191.77	3	< 0.001
Accumulated exposure	direct mortality	0.73	279.18	3	< 0.001
	habitat change	0.58	183.93	3	< 0.001
	climate change	0.80	343.83	3	< 0.001

Table A2 – Outputs of post-hoc tests of multiple comparisons carried out on models assessing the effect of season on mean hourly and accumulated change exposure for each of the three change types: direct mortality, habitat change, climate change, run on a subset of data excluding data-poor bird-seasons.

Model	Pairwise comparison			Estimate	Std. Error	z-value	*P*-value
Mean hourly direct mortality ~ season	**Autumn migration**	**-**	**Breeding**	**1.03**	**0.16**	**6.63**	**<0.001**
	**Spring migration**	**-**	**Breeding**	**1.03**	**0.18**	**5.78**	**<0.001**
	Winter	-	Breeding	0.17	0.17	1.00	0.7500
	Spring migration	-	Autumn migration	0.00	0.16	-0.01	1.0000
	**Winter**	**-**	**Autumn migration**	**-0.86**	**0.15**	**-5.87**	**<0.001**
	**Winter**	**-**	**Spring migration**	**-0.86**	**0.17**	**-5.02**	**<0.001**
Mean hourly habitat change ~ season	Pairwise comparison			Estimate	Std. Error	z-value	*P*-value
	Autumn migration	-	Breeding	-0.09	0.06	-1.41	0.4921
	Spring migration	-	Breeding	-0.15	0.07	-2.02	0.1803
	**Winter**	**-**	**Breeding**	**-2.28**	**0.07**	**-32.61**	**<0.001**
	Spring migration	-	Autumn migration	-0.06	0.07	-0.89	0.8090
	**Winter**	**-**	**Autumn migration**	**-2.19**	**0.06**	**-36.17**	**<0.001**
	**Winter**	**-**	**Spring migration**	**-2.13**	**0.07**	**-30.16**	**<0.001**
Mean hourly climate change ~ season	Pairwise comparison			Estimate	Std. Error	z-value	*P*-value
	**Autumn migration**	**-**	**Breeding**	**1.78**	**0.11**	**16.31**	**<0.001**
	**Spring migration**	**-**	**Breeding**	**1.77**	**0.12**	**14.22**	**<0.001**
	**Winter**	**-**	**Breeding**	**0.57**	**0.12**	**4.84**	**<0.001**
	Spring migration	-	Autumn migration	-0.02	0.11	-0.15	0.9988
	**Winter**	**-**	**Autumn migration**	**-1.21**	**0.10**	**-11.72**	**<0.001**
	**Winter**	**-**	**Spring migration**	**-1.19**	**0.12**	**-9.98**	**<0.001**

Model	Pairwise comparison			Estimate	Std. Error	z-value	*P*-value
Accumulated direct mortality ~ season							
	**Autumn migration**	**-**	**Breeding**	**0.39**	**0.10**	**3.99**	**<0.001**
	**Spring migration**	**-**	**Breeding**	**-0.71**	**0.11**	**-6.28**	**<0.001**
	**Winter**	**-**	**Breeding**	**1.80**	**0.11**	**16.78**	**<0.001**
	**Spring migration**	**-**	**Autumn migration**	**-1.10**	**0.10**	**-11.09**	**<0.001**
	**Winter**	**-**	**Autumn migration**	**1.41**	**0.09**	**15.27**	**<0.001**
	**Winter**	**-**	**Spring migration**	**2.51**	**0.11**	**23.09**	**<0.001**
Accumulated habitat change ~ season	Pairwise comparison			Estimate	Std. Error	z-value	*P*-value
	Autumn migration	-	Breeding	-0.23	0.12	-1.90	0.2271
	**Spring migration**	**-**	**Breeding**	**-1.37**	**0.14**	**-9.94**	**<0.001**
	**Winter**	**-**	**Breeding**	**-1.86**	**0.13**	**-14.17**	**<0.001**
	**Spring migration**	**-**	**Autumn migration**	**-1.15**	**0.12**	**-9.42**	**<0.001**
	**Winter**	**-**	**Autumn migration**	**-1.64**	**0.11**	**-14.42**	**<0.001**
	**Winter**	**-**	**Spring migration**	**-0.49**	**0.13**	**-3.67**	**0.0014**
Accumulated climate change ~ season	Pairwise comparison			Estimate	Std. Error	z-value	*P*-value
	**Autumn migration**	**-**	**Breeding**	**0.72**	**0.08**	**8.53**	**<0.001**
	**Spring migration**	**-**	**Breeding**	**-0.46**	**0.10**	**-4.72**	**<0.001**
	**Winter**	**-**	**Breeding**	**2.08**	**0.09**	**22.40**	**<0.001**
	**Spring migration**	**-**	**Autumn migration**	**-1.18**	**0.09**	**-13.79**	**<0.001**
	**Winter**	**-**	**Autumn migration**	**1.36**	**0.08**	**17.00**	**<0.001**
	**Winter**	**-**	**Spring migration**	**2.54**	**0.09**	**27.02**	**<0.001**

Table A3 – Summary of linear and generalized additive models and associated likelihood ratio tests assessing the influence of autumn flyway longitude on autumn and winter accumulated change exposure scores, run on a subset of data excluding data-poor bird-seasons. Estimated degrees of freedom (edf) are presented for generalized additive models.

Response variable							Likelihood ratio test statistics		
Season	Change type	Model type	AIC	r^2 (adj.)	Sample size	edf	χ^2	χ^2 df	*P*-value
Autumn migration	direct mortality	**GAM**	**227.70**	**0.18**	**84**	**3.05**	**19.78**	**3.05**	**< 0.001**
		linear	235.38	0.08	84	NA	8.00	1.00	0.005
	habitat change	**GAM**	**235.35**	**0.11**	**84**	**3.66**	**13.34**	**3.66**	**0.010**
		linear	242.71	0.00	84	NA	0.67	1.00	0.413
	climate change	**GAM**	**228.48**	**0.16**	**84**	**1.95**	**16.80**	**1.95**	**< 0.001**
		linear	231.05	0.13	84	NA	12.33	1.00	< 0.001
Winter	direct mortality	**GAM**	**144.79**	**0.13**	**52**	**1.57**	**8.90**	**1.57**	**0.012**
		linear	145.27	0.11	52	NA	7.29	1.00	0.007
	habitat change	**GAM**	**129.81**	**0.35**	**52**	**1.91**	**24.58**	**1.91**	**< 0.001**
		linear	131.47	0.32	52	NA	21.09	1.00	< 0.001
	climate change	GAM	148.75	0.05	52	1.00	3.81	1.00	0.051
		linear	148.75	0.05	52	NA	3.81	1.00	0.051

Table A4 – Model summaries of nine univariate models and associated likelihood ratio tests assessing the effect of the mean accumulated change exposure for birds from each of the eleven sites on the mean abundance change per site, run on a subset of data excluding data-deficient bird-seasons.

Predictor variable						Likelihood ratio test statistics			
Season	Change type	Intercept	β	r^2	Sample size	χ^2	χ^2 df	*P*-value	Bonferroni-corrected *P*-value
Autumn migration	direct mortality	-0.19	0.10	0.15	11	1.76	1	0.184	1.000
	habitat change	-0.19	0.08	0.11	11	1.22	1	0.269	1.000
	climate change	-0.19	0.09	0.13	11	1.51	1	0.220	1.000
Winter	direct mortality	-0.19	-0.07	0.08	11	0.87	1	0.351	1.000
	habitat change	-0.19	-0.13	0.27	11	3.38	1	0.066	0.948
	climate change	-0.19	-0.02	0.01	11	0.11	1	0.746	1.000
Spring migration	direct mortality	-0.19	0.09	0.13	10	1.35	1	0.246	1.000
	habitat change	-0.19	0.12	0.22	10	2.49	1	0.115	1.000
	climate change	-0.19	0.00	0.00	10	0.00	1	0.986	1.000
**Breeding**	**direct mortality**	**-0.18**	**-0.22**	**0.72**	**10**	**12.75**	**1**	**0.0004**	**0.024**
	habitat change	-0.18	-0.20	0.59	10	8.99	1	0.003	0.108
	climate change	-0.18	-0.02	0.01	10	0.07	1	0.789	1.000

Winter destination

To explore the extent to which flyway destination affects cumulative winter change exposure, we modelled accumulated winter change exposure as a function of initial winter fix longitude. We used linear models and generalised additive models (thin plate regression splines) with default selection of smoothing parameters to explore whether there was statistical support for non-linear relationships; we used r^2 values and AIC values to compare the fit of the linear and generalised additive models.

Results were very similar to those relating accumulated winter exposure to mid-flyway longitude (Main text). Winter destination longitude predicted accumulated winter exposure to all three change types, with a non-linear relationship between winter destination and accumulated direct mortality and habitat change exposure, and a negative linear relationship between winter destination and accumulated climate change exposure (Table A5, Figure A1).

Table A5 – Summary of linear and generalized additive models and associated likelihood ratio tests assessing the influence of initial winter longitude on subsequent accumulated winter change exposure scores. Models in bold are presented in Figure A1. Estimated degrees of freedom (edf) are presented for generalized additive models.

Response variable							Likelihood ratio test statistics		
	change type	model type	AIC	r^2 (adj.)	sample size	edf	χ^2	χ^2 df	*P*-value
winter	mortality	**GAM**	**154.69**	**0.24**	**58**	**3.40**	**19.70**	**3.40**	**< 0.001**
		linear	159.18	0.15	58	-	10.41	1	0.001
	habitat	**GAM**	**138.50**	**0.42**	**58**	**2.19**	**33.48**	**2.19**	**< 0.001**
		linear	144.95	0.33	58	-	24.63	1	< 0.001
	climate	GAM	162.35	0.10	58	1	7.24	1	0.007
		**linear**	**162.35**	**0.10**	**58**	**-**	**7.24**	**1**	**0.007**

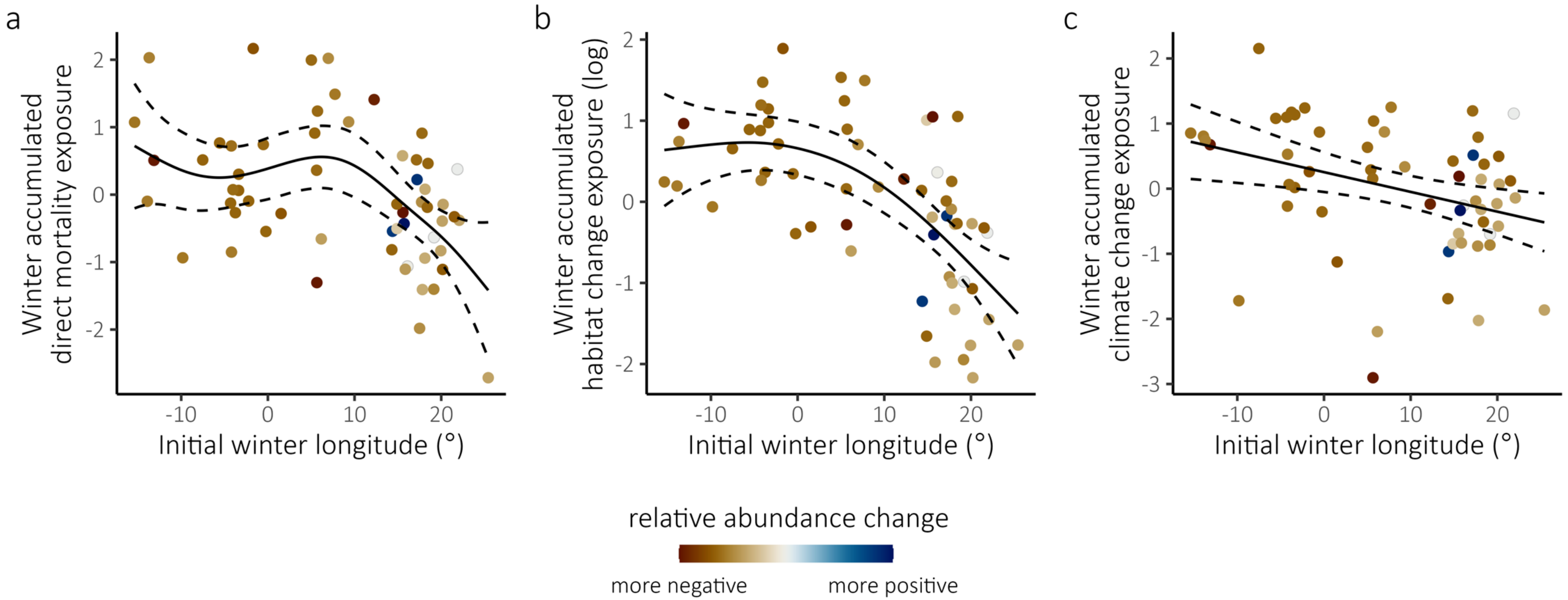

Figure A1 – Plots showing the relationship between initial winter longitude on metrics of accumulated winter change exposure, as predicted by models in bold in Table A5. Points indicate raw data, with each point representing a bird-season. Solid and dashed lines indicate model-predicted means and 95% confidence intervals respectively. Points are coloured according to relative breeding site population abundance change (see Methods).

REFERENCES

Nakagawa, S., & Schielzeth, H. (2013). A general and simple method for obtaining R2 from generalized linear mixed-effects models. *Methods in Ecology and Evolution*, *4*(2), 133–142. https://doi.org/10.1111/j.2041-210x.2012.00261.x
