# Supplementary material for: Combining remote sensing and tracking data to quantify species' cumulative exposure to anthropogenic change
Source: Glob Chang Biol. 2023 Oct 9;29(23):6679–92. doi: 10.1111/gcb.16974 (PMC10946810; doi:10.1111/gcb.16974)
Supplement: Supplementary file 2 — Data S1 [file GCB-29-6679-s002.pdf]

Combining remote sensing and tracking data to quantify species' cumulative exposure to anthropogenic change

Claire Buchan*, James J. Gilroy, Inês Catry, Chris M. Hewson, Philip W. Atkinson, Aldina M. A. Franco

Supplementary Materials

This document contains supplementary materials relating to the methods and results reported in the above manuscript, and consists of the following materials:

Figure S1 Schematic illustrating how the stages of the annual cycle were defined

Section S1 Description of methods and formulae for combining constituent anthropogenic change layers

Table S1 Summary statistics describing final tracking dataset filtered and categorised into seasons

Table S2 Post-hoc tests of multiple comparisons carried out on models assessing effect of season on mean hourly and accumulated change exposure

Table S3 Summaries of linear and generalized additive models assessing the influence of autumn flyway longitude on autumn and winter accumulated change exposure

Figure S2 Common cuckoo population abundance change maps

Figure S3 Model-predicted relationship between mean breeding season direct mortality change exposure and breeding site population change

Figure S4 Autumn migration and wintering tracks of common cuckoo by breeding population

Figure S5 Relationship between metrics of raw mean hourly change exposure and autumn flyway longitude

References cited

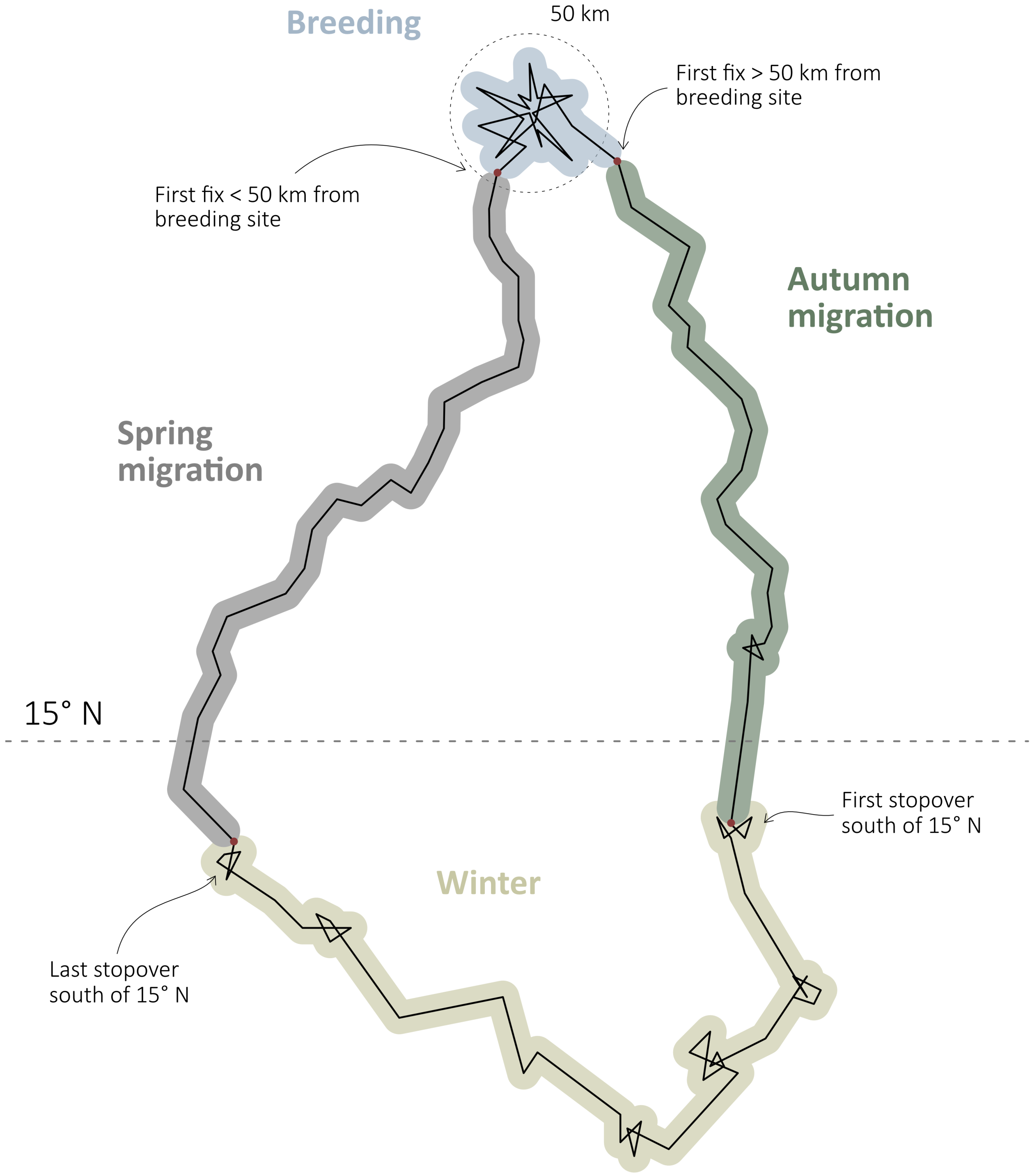

Figure S1 – Schematic illustrating the geographic and behavioural criteria by which we defined the four stages of the annual cycle of the cuckoo. Winter refers to that of the Northern Hemisphere.

Section S1

The following is adapted from Buchan et al. (2022):

Composite mapping is complicated by the possibility that risks associated with exposure to certain change layers might be increasive but non-additive, meaning that the presence of multiple spatially contiguous change metrics may increase the total potential risk, but to a lesser degree than would be implied by direct summation of values (Kennedy et al., 2019). To account for this, we grouped change layers whose impacts were likely to be correlative or non-independent (e.g., human population density, roads and urbanization), and combined them using fuzzy algebraic sums (Theobald, 2013). The fuzzy algebraic sum of a set of values between 0 and 1 is given by 1 minus the product of (1 – *x*), where *x* is each member of the set, such that the final fuzzy summed value is less than the literal sum of its parts, and tends towards a maximum value of 1 (Bonham-Carter, 1994). In cases where change metrics were independent and therefore truly additive (e.g., threat posed by hunting pressure) we used simple linear summation.

While in Buchan et al. 2022, per-species susceptibility weightings are used to create relative vulnerability scores, these are not used here as we are quantifying exposure for a single species; the formulae are adapted/simplified accordingly:

In all cases, where i is a cell:

$$Direct\ mortality_i = [1 - \prod_{j=1}^{5}(1 - M_{i,j})] + \frac{N_i}{n(j)} + \frac{H_i}{n(j)}$$

Where j ($1 \leq j \leq 5$) indicates one of five non-independent direct mortality layers: {urbanisation, population density, roads, windfarms, powerlines}. $M_{i,j}$ is therefore the value for change layer j in cell i. N_i and H_i are, respectively, the nocturnal lights layer and the hunting value for small-bodied bird species in cell i. $n(j)$ denotes the number of layers within j, in this case five.

$$Habitat\ change_i = \left\{1 - [1 - \sum_{h=1}^{2}(A_{i,h})][1 - \frac{F_i}{n(h)}]\right\}$$

Where h ($1 \leq h \leq 2$) indicates one of the two independent habitat layers: {pesticides, urbanisation}, with $A_{i,h}$ being the value for layer h in cell i. F_i is fertilizer in cell i. $n(h)$ denotes the number of layers within h, in this case two.

$$Climate\ change_{m,i} = [2 - (1 - P_{m,i})(1 - V_{m,i}) - (1 - T_{m,i})(1 - Y_{m,i})]$$

Where $P_{m,i}$ is anomaly in precipitation in cell i during month m, $V_{m,i}$ is anomaly in precipitation variability, $T_{m,i}$ is anomaly in temperature and $Y_{m,i}$ is anomaly in temperature variability.

Table S1 – Summary statistics describing final tracking dataset filtered and categorised into seasons

	Total			Mean ±SD				
Season	Bird-Years	Individuals	Fixes	Fixes bird-year^{-1}	Duration (days)	Distance (km)	Start date	End date
Autumn migration	86	53	1937	22.5<br>±8.6	51.0<br>±19	5522<br>±755	21 Jun<br>±10 days	11 Aug<br>±21 days
Winter	58	37	4700	81.0<br>±19.6	229.5<br>±24	6042<br>±1524	12 Aug<br>±20 days	29 Mar<br>±15 days
Spring migration	51	32	481	9.4<br>±3.3	26.5<br>±13	5627<br>±815	03 Apr<br>±14 days	30 Apr<br>±8 days
Breeding	44	26	1054	24.0<br>±8.7	42.9<br>±14	127.1<br>±91	01 May<br>±8 days	13 Jun<br>± 12 days

Section S2

The following describes the model syntax for each of the analyses in the manuscript.

**Between-season exposure:**	**Model type**	**Relevant tables/figures**
Mean direct mortality change exposure (log) ~ season + (1|birdID)	Linear mixed effects	Figure 3; Tables 1 and S2
Mean habitat change exposure (log) ~ season + (1|birdID)	Linear mixed effects	Figure 3; Tables 1 and S2
Mean climate change exposure (log) ~ season + (1|birdID)	Linear mixed effects	Figure 3; Tables 1 and S2
**Migratory route:**		
Accumulated autumn direct mortality change exposure (log) ~ longitude at 35° N	Linear and additive	Figure 4; Table S3
Accumulated autumn habitat change exposure (log) ~ longitude at 35° N	Linear and additive	Figure 4; Table S3
Accumulated autumn climate change exposure (log) ~ longitude at 35° N	Linear and additive	Figure 4; Table S3
Accumulated winter direct mortality change exposure ~ longitude at 35° N	Linear and additive	Figure 4; Table S3
Accumulated winter habitat change exposure (log) ~ longitude at 35° N	Linear and additive	Figure 4; Table S3
Accumulated winter climate change exposure ~ longitude at 35° N	Linear and additive	Figure 4; Table S3
Accumulated winter direct mortality change exposure ~ longitude at first winter fix	Linear and additive	Figure A1; Table A5
Accumulated winter habitat change exposure (log) ~ longitude at first winter fix	Linear and additive	Figure A1; Table A5
Accumulated winter climate change exposure ~ longitude at first winter fix	Linear and additive	Figure A1; Table A5

Breeding site abundance change:

Mean site abundance change ~ accumulated autumn direct mortality change (log)	Linear	Figure S3; Table 2
Mean site abundance change ~ accumulated autumn habitat change (log)	Linear	Figure S3; Table 2
Mean site abundance change ~ accumulated autumn climate change (log)	Linear	Figure S3; Table 2
Mean site abundance change ~ accumulated winter direct mortality change	Linear	Figure S3; Table 2
Mean site abundance change ~ accumulated winter habitat change	Linear	Figure S3; Table 2
Mean site abundance change ~ accumulated winter climate change (cubed)	Linear	Figure S3; Table 2
Mean site abundance change ~ accumulated spring direct mortality change (log)	Linear	Figure S3; Table 2
Mean site abundance change ~ accumulated spring habitat change (log)	Linear	Figure S3; Table 2
Mean site abundance change ~ accumulated spring climate change	Linear	Figure S3; Table 2
Mean site abundance change ~ accumulated breeding direct mortality change (cubed)	Linear	Figure S3; Table 2
Mean site abundance change ~ accumulated breeding habitat change (log)	Linear	Figure S3; Table 2
Mean site abundance change ~ accumulated breeding climate change (log)	Linear	Figure S3; Table 2

Table S2 – Outputs of post-hoc tests of multiple comparisons carried out on models assessing the effect of season on mean hourly and accumulated change exposure for each of the three change types: direct mortality, habitat change, climate change.

Model	Pairwise comparison			Estimate	Std. Error	z-value	*P*-value
Mean hourly direct mortality ~ season							
	**Autumn migration**	**-**	**Breeding**	**0.87**	**0.16**	**5.59**	**<0.001**
	**Spring migration**	**-**	**Breeding**	**1.20**	**0.17**	**7.06**	**<0.001**
	Winter	-	Breeding	0.17	0.17	1.00	0.751
	Spring migration	-	Autumn migration	0.33	0.15	2.25	0.108
	**Winter**	**-**	**Autumn migration**	**-0.70**	**0.14**	**-4.97**	**<0.001**
	**Winter**	**-**	**Spring migration**	**-1.04**	**0.16**	**-6.53**	**<0.001**
Mean hourly habitat change ~ season	Pairwise comparison			Estimate	Std. Error	z-value	*P*-value
	Autumn migration	-	Breeding	-0.10	0.07	-1.58	0.388
	Spring migration	-	Breeding	-0.13	0.07	-1.84	0.252
	**Winter**	**-**	**Breeding**	**-2.24**	**0.07**	**-31.74**	**<0.001**
	Spring migration	-	Autumn migration	-0.03	0.06	-0.46	0.967
	**Winter**	**-**	**Autumn migration**	**-2.14**	**0.06**	**-35.50**	**<0.001**
	**Winter**	**-**	**Spring migration**	**-2.11**	**0.07**	**-31.22**	**<0.001**
Mean hourly climate change ~ season	Pairwise comparison			Estimate	Std. Error	z-value	*P*-value
	**Autumn migration**	**-**	**Breeding**	**1.49**	**0.12**	**12.14**	**<0.001**
	**Spring migration**	**-**	**Breeding**	**1.78**	**0.13**	**13.35**	**<0.001**
	**Winter**	**-**	**Breeding**	**0.53**	**0.13**	**4.03**	**<0.001**
	Spring migration	-	Autumn migration	0.29	0.12	2.53	0.0551
	**Winter**	**-**	**Autumn migration**	**-0.96**	**0.11**	**-8.63**	**<0.001**
	**Winter**	**-**	**Spring migration**	**-1.26**	**0.12**	**-10.09**	**<0.001**

Model	Pairwise comparison			Estimate	Std. Error	z-value	*P*-value
Accumulated direct mortality ~ season	Pairwise comparison			Estimate	Std. Error	z-value	*P*-value
	**Autumn migration**	**-**	**Breeding**	**0.37**	**0.10**	**3.67**	**0.00138**
	**Spring migration**	**-**	**Breeding**	**-0.60**	**0.11**	**-5.32**	**<0.001**
	**Winter**	**-**	**Breeding**	**1.79**	**0.11**	**16.43**	**<0.001**
	**Spring migration**	**-**	**Autumn migration**	**-0.96**	**0.10**	**-10.04**	**<0.001**
	**Winter**	**-**	**Autumn migration**	**1.42**	**0.09**	**15.33**	**<0.001**
	**Winter**	**-**	**Spring migration**	**2.38**	**0.10**	**22.82**	**<0.001**
Accumulated habitat change ~ season	Pairwise comparison			Estimate	Std. Error	z-value	*P*-value
	Autumn migration	-	Breeding	-0.26	0.12	-2.09	0.15513
	**Spring migration**	**-**	**Breeding**	**-1.36**	**0.14**	**-9.97**	**<0.001**
	**Winter**	**-**	**Breeding**	**-1.80**	**0.13**	**-13.56**	**<0.001**
	**Spring migration**	**-**	**Autumn migration**	**-1.10**	**0.12**	**-9.35**	**<0.001**
	**Winter**	**-**	**Autumn migration**	**-1.54**	**0.11**	**-13.65**	**<0.001**
	**Winter**	**-**	**Spring migration**	**-0.44**	**0.13**	**-3.47**	**0.00272**
Accumulated climate change ~ season	Pairwise comparison			Estimate	Std. Error	z-value	*P*-value
	**Autumn migration**	**-**	**Breeding**	**0.70**	**0.09**	**8.04**	**<0.001**
	**Spring migration**	**-**	**Breeding**	**-0.35**	**0.10**	**-3.59**	**0.00179**
	**Winter**	**-**	**Breeding**	**2.07**	**0.09**	**21.96**	**<0.001**
	**Spring migration**	**-**	**Autumn migration**	**-1.05**	**0.08**	**-12.61**	**<0.001**
	**Winter**	**-**	**Autumn migration**	**1.36**	**0.08**	**17.07**	**<0.001**
	**Winter**	**-**	**Spring migration**	**2.41**	**0.09**	**26.73**	**<0.001**

Table S3 – Summary of linear and generalized additive models and associated likelihood ratio tests assessing the influence of autumn flyway longitude on autumn and winter accumulated change exposure scores. Models in bold are presented in Figure 3. Estimated degrees of freedom (edf) are presented for generalized additive models.

Response variable							Likelihood ratio test statistics		
Season	Change type	Model type	AIC	r^2 (adj.)	Sample size	edf	χ^2	χ^2 df	*P*-value
Autumn migration	direct mortality	**GAM**	**238.49**	**0.12**	**86**	**2.75**	**14.05**	**2.75**	**0.003**
		linear	243.41	0.05	86	-	5.64	1.00	0.018
	habitat change	**GAM**	**244.86**	**0.06**	**86**	**3.23**	**8.65**	**3.23**	**0.034**
		linear	248.97	-0.01	86	-	0.08	1.00	0.782
	climate change	**GAM**	**237.37**	**0.13**	**86**	**1.94**	**13.56**	**1.94**	**0.001**
		linear	239.69	0.09	86	-	9.36	1.00	0.002
Winter	direct mortality	**GAM**	**158.50**	**0.18**	**58**	**2.56**	**14.21**	**2.56**	**0.003**
		linear	160.77	0.13	58	-	8.82	1.00	0.003
	habitat changes	**GAM**	**141.19**	**0.38**	**58**	**1.65**	**29.70**	**1.65**	**< 0.001**
		linear	141.99	0.37	58	-	27.59	1.00	<0.001
	climate change	GAM	164.32	0.07	58	1.00	5.27	1.00	0.02
		**linear**	**164.32**	**0.07**	**58**	**-**	**5.27**	**1.00**	**0.02**

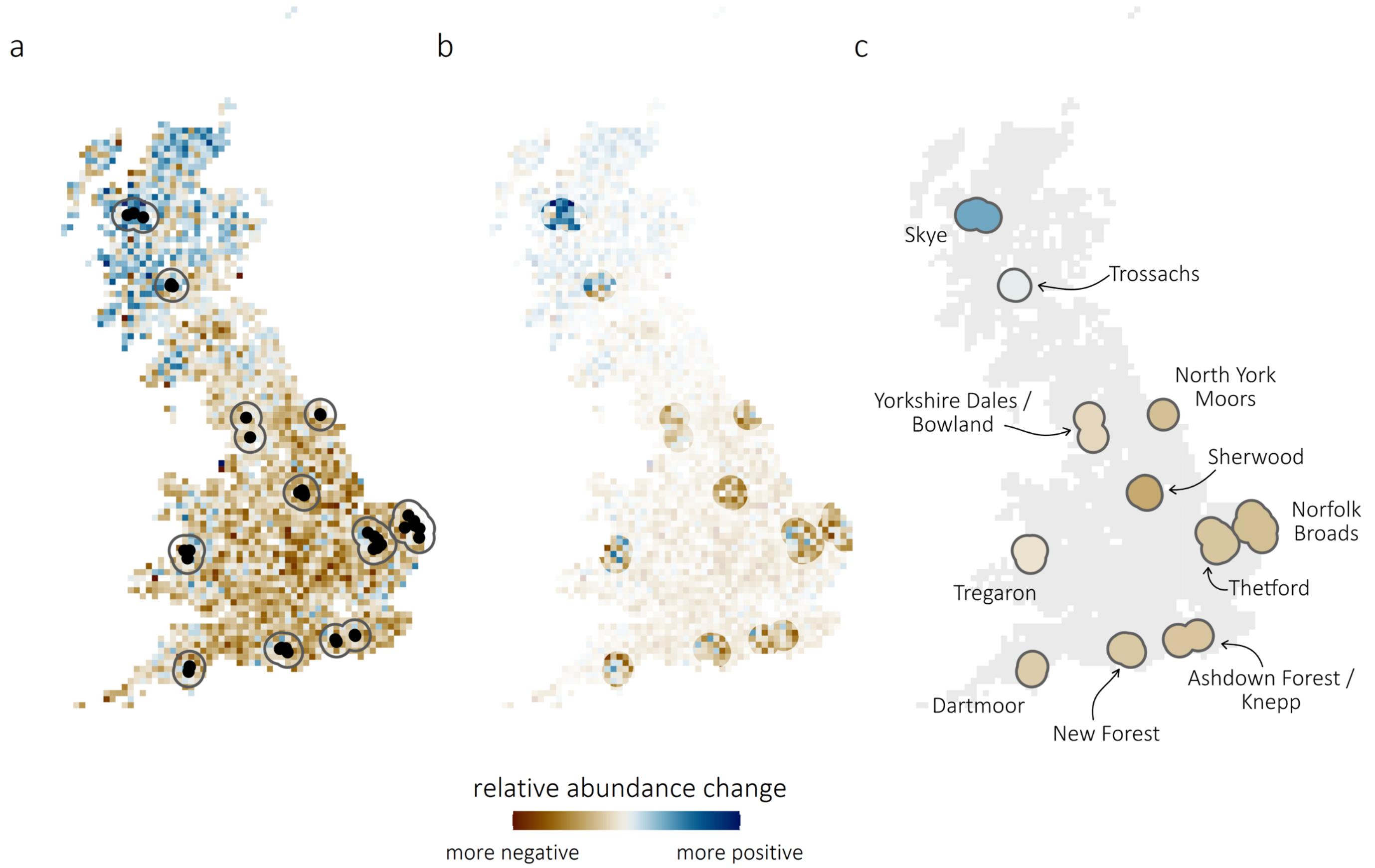

Figure S2 – a: Standardised population abundance change of common cuckoos between 1988–1992 and 2007–11 (Balmer et al., 2014) at a 10 km x 10km resolution. Black points indicate capture locations of each individual included within this analysis. Grey rings indicate the site region defined by a 25-km radius buffer around the capture locations. b: Standardised population abundance change included in the calculation of each site mean. c: Location of each site and relative spatial mean population abundance change.

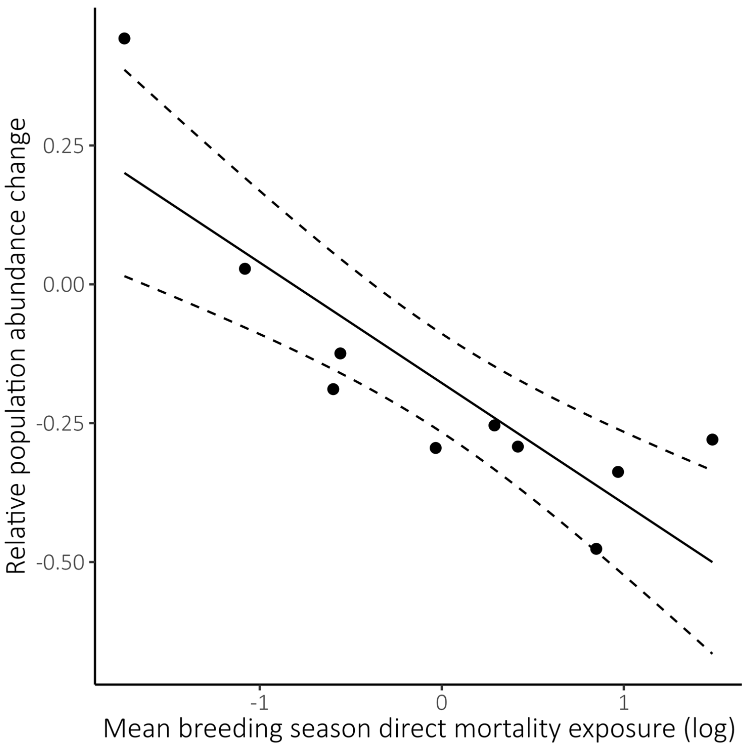

Figure S3 – Plot showing the relationship between mean breeding season mortality change exposure and breeding site relative population change. Points indicate raw data, with each point representing a UK breeding site. Solid and dashed lines indicate the mean and associated 95% confidence intervals respectively as predicted by the model in Table 2 of the main manuscript.

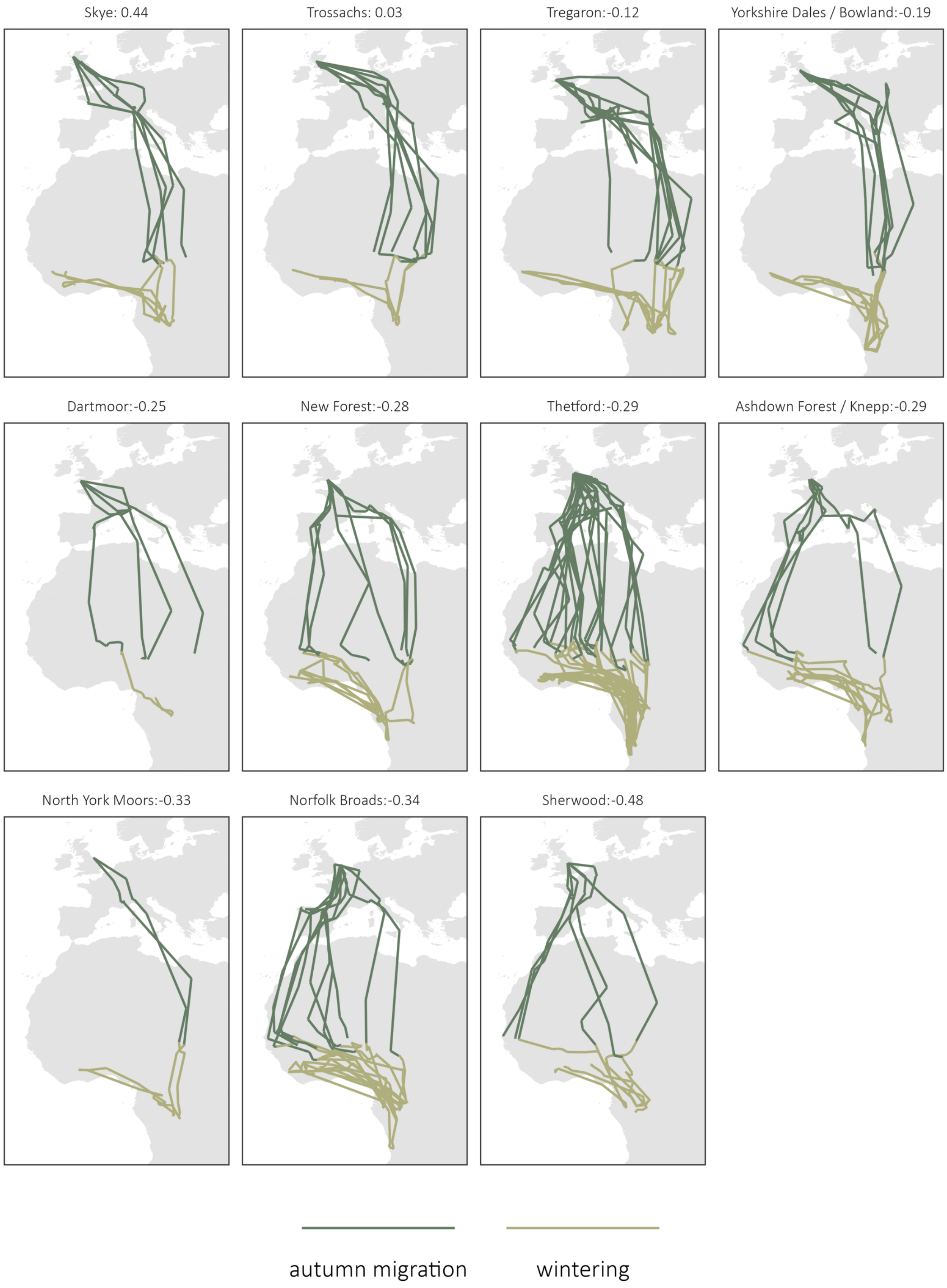

Figure S4 – Autumn migration (green) and wintering tracks (yellow) of tagged common cuckoos from eleven different breeding populations within the UK. The variability of routes taken by individuals from within a breeding site indicate weak migratory connectivity at this scale. Values at the top of each panel are the relative population abundance change (see Methods).

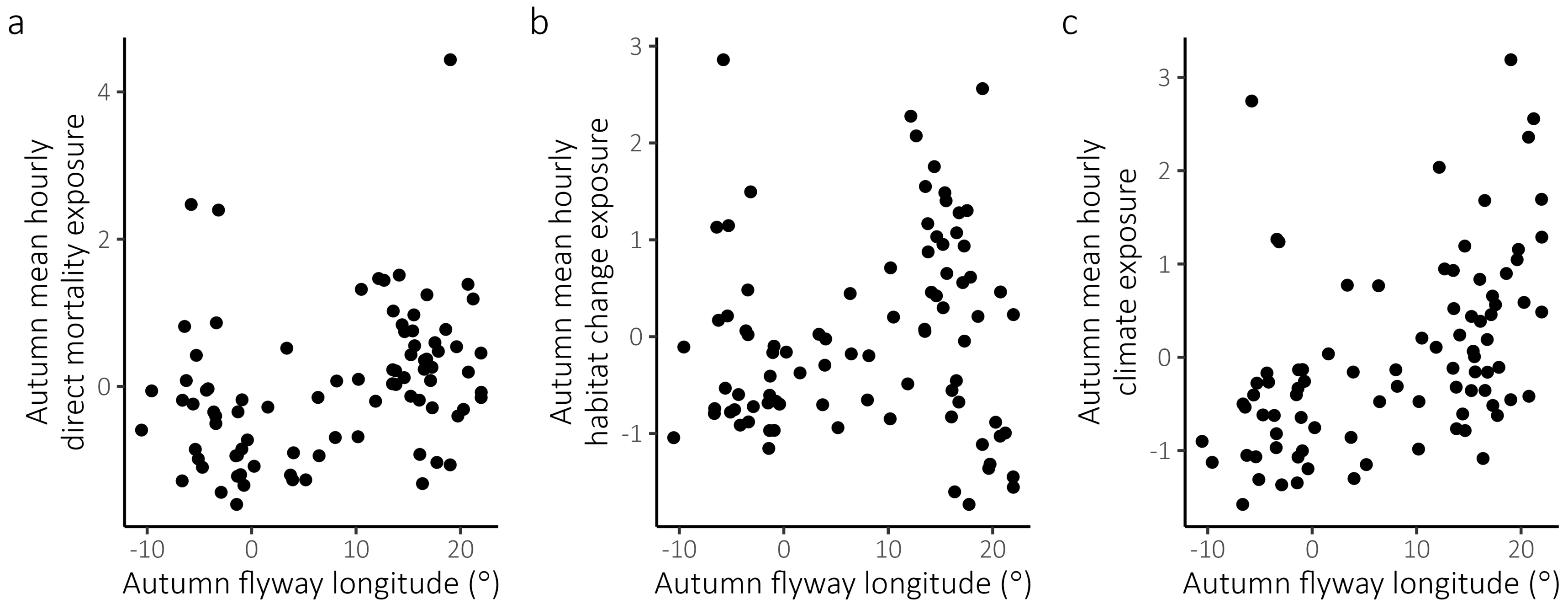

Figure S5 – Scatterplots showing the relationship between mean hourly direct mortality (a), habitat change (b) and climate change (c) exposure in the autumn migration and autumn migratory route longitudes. Points are raw data, with each point representing a bird-season.

REFERENCES

Balmer, D., Gillings, S., Caffrey, B., Swann, B., Downie, I., & Fuller, R. (2014). *Bird Atlas 2007-11: The Breeding and Wintering Birds of Britain and Ireland*. HarperCollins UK.

Bonham-Carter, G. F. (1994). *Geographic Information Systems for Geoscientists: Modelling with GIS*. Elsevier.

Buchan, C., Franco, A. M. A., Catry, I., Gamero, A., Klvaňová, A., & Gilroy, J. J. (2022). Spatially explicit risk mapping reveals direct anthropogenic impacts on migratory birds. *Global Ecology and Biogeography*, *31*(9), 1707–1725. https://doi.org/10.1111/geb.13551

Kennedy, C. M., Oakleaf, J. R., Theobald, D. M., Baruch-Mordo, S., & Kiesecker, J. (2019). Managing the middle: A shift in conservation priorities based on the global human modification gradient. *Global Change Biology*, *25*(3), 811–826. https://doi.org/10.1111/gcb.14549

Theobald, D. M. (2013). A general model to quantify ecological integrity for landscape assessments and US application. *Landscape Ecology*, *28*(10), 1859–1874. https://doi.org/10.1007/s10980-013-9941-6
